# Successful awake craniotomy in an aged patient with a severe hearing impairment using a bone conduction voice amplifier: a case report

**DOI:** 10.1186/s40981-019-0258-6

**Published:** 2019-06-07

**Authors:** Shunsuke Tachibana, Masahito Omote, Michiaki Yamakage

**Affiliations:** Department of Anesthesiology, School of Medicine, Sapporo Medical University, South 1, West 16, Chuo-ku, Sapporo, Hokkaido 060-8543 Japan

**Keywords:** Awake craniotomy, Severe hearing impairment, Bone conduction voice amplifier

## Abstract

**Background:**

The main purposes of awake craniotomy are to minimize postoperative brain dysfunction caused by the surgical procedure and to maximize the tumor resection range. In awake craniotomy, it is important to have a good quality of awakening and to obtain patient’s obedience in the awake phase.

**Case presentation:**

The patient was a 75-year-old woman with an advanced hearing impairment who was scheduled for awake craniotomy. We used a bone conduction voice amplifier before and during the awake phase and communicated with the patient smoothly.

**Conclusions:**

We were able to complete awake craniotomy fully, and overcoming the deafness problem might have contributed to the patient’s good outcome. This case report indicates that awake craniotomy can be performed in a patient with an advanced hearing impairment under the condition of careful anesthetic management.

## Background

The primary objective of awake craniotomy (AC) is to remove as much of the brain tumor as possible and to minimize postoperative brain dysfunction. The asleep-awake-asleep (AAA) method or the monitored anesthetic care (MAC) method is used for anesthetic management in AC [1]. In either anesthesia method, the patient is managed in an awake state during mapping by electrical stimulation and monitoring the neurological findings. Therefore, it is essential to maintain sufficient quality of the patient’s awareness and to control the patient’s obedience, especially in the AAA method. Several indications for AC are documented in guidelines [2].

In this case report, we present a case of severe hearing impairment in an aged patient in whom AC was successfully performed with smooth communication by using a bone conduction voice amplifier.

## Case presentation

We obtained written informed consent for this case report from the patient. A 75-year-old woman (height 160 cm, weight 45 kg, BMI 17.6 kg/m^2^) who was diagnosed with a large glioma. The tumor was a maximum diameter of 65 mm and was spread within and near the right primary motor area, which is categorized as an eloquent area. She was scheduled to undergo AC for tumor resection; however, she had a severe hearing impairment (both audiometric levels in decibels being around 70) because of advanced age and a history of myringoplasty. For anesthetic management, we selected the asleep-awake-asleep method, which is the usual protocol in our hospital.

**Fig. 1:**
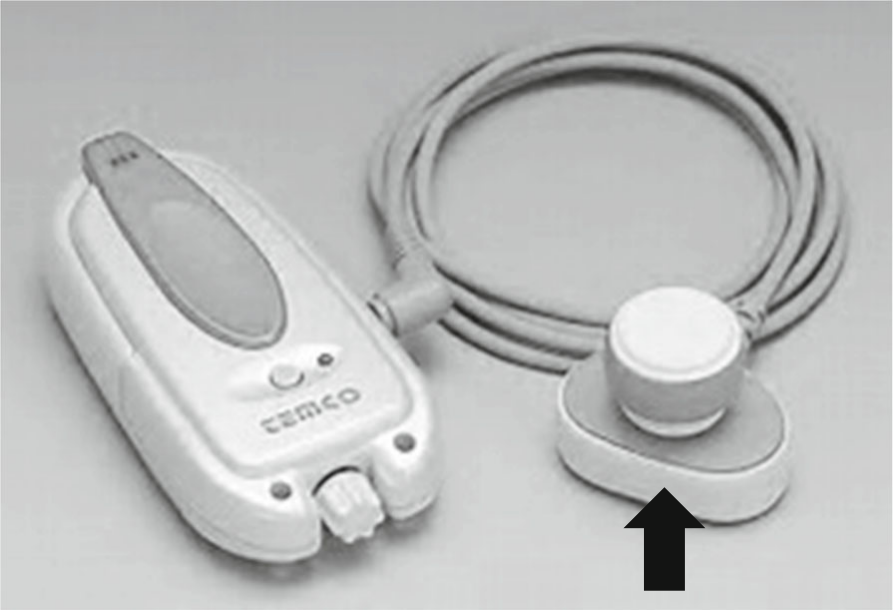
A bone conduction voice amplifier used in this case. This device was authorized as a medical device (authorization number is 21300BZY00587000). The part that the black arrow shows was applied to the back of the patient’s ear

Anesthesia was induced with propofol and remifentanil, and LMA Supreme® was inserted smoothly after administration of rocuronium. In order to prevent cranial pain during the awake phase, cranial nerve blocks and infiltration anesthesia for the scalp incision were performed by using 0.375% levobupivacaine. The surgery was started with the patient in a lateral position. Propofol and remifentanil were used for maintenance of anesthesia, but the doses of these drugs were gradually decreased toward the awake phase. When the brain surgeons gave the cue, we stopped administration of the drugs and awakened the patient. Just before awakening, a bone conduction voice amplifier (HA301-K “Kikuchan”, Temco Japan Co., Ltd., Tokyo; shown in Fig. 1) was attached to the patient behind the left ear auricle on the non-operative side, and we called the patient’s name until she awakened. It took 15 min from the awakening cue to LMA extubation and a further 10 min until definite awakening. By using the bone conduction voice amplifier, the anesthesiologist’s instructions could be transmitted to the patient, and there were no problems in subsequent responses. We instructed the patient to move her upper and lower left limbs in order to monitor the motor area in the vicinity of the tumor removal, and there was no dropout in obedience and movements. The surgery was completed without adverse events including intraoperative cranial pain, nausea and vomiting, and seizures.

The awake phase time was 123 min, total operation time was 484 min, and total anesthesia time was 621 min. We did not observe the transient or permanent deterioration of motor function after surgery.

## Discussion

The purposes of AC are to remove as much of the tumor as possible and minimize brain dysfunction resulting from the brain tumor removal [3, 4]. Therefore, during brain mapping and monitoring, it is important to maintain a good quality of awakening in the awake phase. In the guidelines for AC, it is stated that “if the required tasks can be handled correctly, awake surgery can be performed in persons older than 65 years of age.” Regarding neurological symptoms, it is stated in the guidelines that “the patient must be able to recognize whether or not the patient can tolerate awake anesthesia.” [1].

Our patient was an elderly patient with a severe hearing impairment, and it was not clear whether AC was appropriate or not. We have another anesthetic option, general anesthesia with using motor evoked potentials (MEPs). However, the patient strongly desired for awake surgery, and we concluded that the patient’s obedience could be achieved if the hearing impairment problem was overcome. The patient’s preoperative cognitive function was normal (verbal I.Q. 110, performance I.Q. 103, full-scale I.Q. 108), and communication with the patient could be performed smoothly if she could hear the anesthesiologist’s instructions. Using intraoperative MEPs has been proved to be sufficient to detect the deterioration of motor function; however, there are several limitations in clinical use. First, MEPs may be insufficiently sensitive for evaluating voluntary movement under certain conditions. Suzuki et al. reported that there is a false-positive or false-negative case in evaluating with MEPs, and it should be considered that there is a discrepancy between voluntary movement and results of MEPs [5]. Second, additional costs and human resources will be required for setting MEPs.

There has been no previous report on AC being performed with the use of a bone conduction voice amplifier. The bone conduction amplifier used in this case (authorized as a medical device, approval number: 21300BZY00587000) converts the input voice signal into bone-conducted ultrasound and directly transmits to the auditory nerve. A rechargeable lithium battery is built in the body, and there is no interference with other devices. The usefulness of the prototype device has already been proved for hearing impairment [6]. This device is applied to the skull around the ear, and safety in use is also secured.

Using the hearing aid that the patient daily used would have the risk of contamination due to disinfection of the surgery area, breakage, and instability of fixation to the ear canal. Thus, we decided to select a bone condition amplifier. Besides, in hearing impairment cases, it is thought that a bone conduction amplifier can be used not only with the MAC method but also with the AAA method.

The surgery, in this case, might be challenging; however, we were able to perform the management of the patient successfully. In anesthetic management for AC, anesthesiologists often need to consider the diversity of patients’ backgrounds and individual differences. Considering the patient’s wish and prognosis, AC should not be rejected simply because the patient has a hearing impairment.

By using this device properly, it was possible to maintain the awakening state and to obtain sufficient obedience from the patient without noticeable complications. Using a bone conduction voice amplifier would enable AC to be completed in this case.

## Conclusions

Even in cases of severe hearing impairment, AC can be completed by using a bone conduction voice amplifier. Overcoming the problem by using such a device might contribute to a good outcome.

## Data Availability

Not applicable

## Abbreviations

AAA: Asleep-awake-asleep
AC: Awake craniotomy
BMI: Body mass index
I.Q.: Intelligence quotient
MAC: Monitored anesthetic care
MEPs: Motor evoked potentials
